# Immune State Conversion of the Mesenteric Lymph Node in a Mouse Breast Cancer Model

**DOI:** 10.3390/ijms231911035

**Published:** 2022-09-20

**Authors:** Tsukasa Shigehiro, Maho Ueno, Mayumi Kijihira, Ryotaro Takahashi, Chiho Umemura, Eman A. Taha, Chisaki Kurosaka, Megumi Asayama, Hiroshi Murakami, Ayano Satoh, Yoshimasa Nakamura, Junichiro Futami, Junko Masuda

**Affiliations:** 1 Research Institute for Biomedical Sciences, Tokyo University of Science, Chiba 278-0022, Japan; 2 Center for Immunotherapy, Roswell Park Comprehensive Cancer Center, Buffalo, NY 14263, USA; 3 Department of Applied Chemistry and Biotechnology, Faculty of Engineering, Okayama University, Okayama 700-8530, Japan; 4 Graduate School of Interdisciplinary Science and Engineering in Health Systems, Okayama University, Okayama 700-8530, Japan; 5 Division of Medical Bioengineering, Graduate School of Natural Science and Technology, Okayama University, Okayama 700-8530, Japan; 6 Graduate School of Environmental and Life Science, Okayama University, Okayama 700-8530, Japan; 7 Department of Pharmacology, Tokyo Women’s Medical University, Shinjuku, Tokyo 162-8666, Japan

**Keywords:** breast cancer cells, dendritic cells, mesenteric lymph node, myeloid-derived suppressor cells

## Abstract

Secondary lymphoid tissues, such as the spleen and lymph nodes (LNs), contribute to breast cancer development and metastasis in both anti- and pro-tumoral directions. Although secondary lymphoid tissues have been extensively studied, very little is known about the immune conversion in mesenteric LNs (mLNs) during breast cancer development. Here, we demonstrate inflammatory immune conversion of mLNs in a metastatic 4T1 breast cancer model. Splenic T cells were significantly decreased and continuously suppressed IFN-γ production during tumor development, while myeloid-derived suppressor cells (MDSCs) were dramatically enriched. However, T cell numbers in the mLN did not decrease, and the MDSCs only moderately increased. T cells in the mLN exhibited conversion from a pro-inflammatory state with high IFN-γ expression to an anti-inflammatory state with high expression of IL-4 and IL-10 in early- to late-stages of breast cancer development. Interestingly, increased migration of CD103^+^CD11b^+^ dendritic cells (DCs) into the mLN, along with increased (1→3)-β-D-glucan levels in serum, was observed even in late-stage breast cancer. This suggests that CD103^+^CD11b^+^ DCs could prime cancer-reactive T cells. Together, the data indicate that the mLN is an important lymphoid tissue contributing to breast cancer development.

## 1. Introduction

Transformed cells with mutations in cancer-related genes are associated with uncontrolled cell division and are deficient in the repair of gene replication errors. During cancer initiation and development, abnormal expression of various genes also leads to the secretion of cytokines and growth factors inappropriate for the cellular environment causing decay of the surroundings. This facilitates the development of a tumor microenvironment with many stromal cells such as myofibroblasts, vascular endothelial cells, pericytes, and immune cells [1,2,3]. Immune cells such as dendritic cells, macrophages, and CD4^+^ and CD8^+^ T cells can potentially play a role in antitumor immunity in the tumor milieu [4]. However, antitumor immunity is attenuated by factors such as increases in myeloid-derived suppressor cells (MDSCs) and CD4^+^ regulatory T cells (Tregs), as well as the reduction in cytotoxic T lymphocyte function responsible for directly killing cancer cells [3,5,6,7,8,9,10,11,12]. Furthermore, the increase in MDSCs reduces the number of CD4^+^ and CD8^+^ T cells and further modulates the expression of cytokines such as IFN-γ, IL-4, and IL-10 in the spleen [5,6]. Thus, tumor proliferation has a major impact on the immune cells located in the tumor microenvironment, as well as on systemic immunity.

The intestinal tract hosts the highest number of immune cells in the body, maintaining a fine-tuned balance between inflammatory responses to potential pathogens and tolerance to commensal bacteria or food antigens [13]. The intestine consists of several functionally specialized cells in the epithelia and lamina propria, and regulates its immunological homeostasis independently of systemic immunity [14]. However, recent findings suggest that mucosal tissue could interact with other organs via immune cells. For example, CD4^+^ T cells activated in the lungs due to an influenza infection migrate into intestinal tract and cause T helper (Th)-17-mediated intestinal inflammation, which is accompanied by a change in commensal bacteria population [15,16]. In addition, activation of toll-like receptor (TLR)-5-dependent signaling induced by intestinal bacteria contributes to tumor malignancy in mouse models [17]. However, whether the disturbance caused in systemic immune cells by tumor progression at sites other than the intestine influences the intestinal immune homeostasis is not yet known.

Breast cancer is the most common malignant disease in women and often metastasizes to the lymph nodes, lungs, and bones [18]. Similarly to human breast cancer cells, the BALB/c-derived breast cancer cell line 4T1 cells have the ability to actively metastasize to the lungs, liver, bone, and spleen, and occasionally metastasize to tumor-draining lymph nodes, the brain, intestine, kidney, and adrenals [19,20]. Murine subcutaneous tumor models with normal immune function have been reported to show the highest accumulation of splenic MDSCs due to tumor progression compared to other syngeneic tumor-bearing mice [21]. However, whether intestinal immune cells are influenced by breast cancer progression is currently unknown.

In the present study, we utilized a syngeneic mouse breast cancer model, and investigated the changes in immune cells in the mesenteric lymph node (mLN) and spleen in a late stage of breast cancer to explore the association between intestinal immune cells and cancer.

## 2. Materials and Methods

### 2.1. Reagents and Monoclonal Antibodies (mAbs)

Anti-CD3ε (145-2C11), CD11b (M1/70), and CD103 (M290) mAbs were purchased from BD Biosciences (San Jose, CA, USA). Anti-CD25 (PC61.5), Gr-1 (RB6-8C5), and CD16/CD32 (93) mAbs were purchased from eBioscience (San Diego, CA, USA). Anti-CD4 (RM4–5), CD8α (53–6.7), CD11c (N148), and MHC-class II (M5/114.15.2) were purchased from Tonbo Biosciences (San Diego, CA, USA).

### 2.2. Animals

Female BALB/c mice (age: 5 weeks; weight: 17–19 g) were obtained from Charles River Inc. (Kanagawa, Japan) and maintained under specific pathogen-free conditions at the Tsushima-kita Branch, Department of Animal Resources, Advanced Research Center, Okayama University. The animals were kept at 22–26 °C and under 50% humidity with a 12-h light/dark cycle and were fed a standardized diet with ad libitum access to autoclaved tap water.

### 2.3. Tumor Cell Cultures

The mammary carcinoma cell line 4T1-Luc (JCRB1447, the Japanese Cancer Research Resources Bank, Ibaraki, Japan) was cultured in Roswell Park Memorial Institute (RPMI) 1640 medium (Sigma-Aldrich, St. Louis, MO, USA) supplemented with 10% (*v/v*) heat-inactivated fetal bovine serum (FBS) (SAFC Biosciences, Lenexa, KS, USA) and 1% (*v/v*) antibiotic-antimycotic solution (10,000 U/mL penicillin, 10,000 μg/mL streptomycin, and 25 μg/mL amphotericin B; Life Technologies, Gaithersburg, MD, USA).

### 2.4. Tumor Cell Implantations

The 4T1 cells (5 × 10^5^ cells/200 µL/mouse) were implanted subcutaneously into the right flank of a 6-week-old mouse. A sham mouse injected with saline only served as a control. The mice were euthanized at days 14 and 21, and the tissue samplings were conducted accordingly.

### 2.5. Splenocyte and mLN Cultures and the Assays for Cytokine Levels

Splenocytes and mLN mononuclear cells were isolated and cultured as previously described [5,6,7,22,23]. Briefly, the immune cells from the spleen and mLN (4 × 10^5^ cells/well) were cultured for 48 h on flat-bottomed 96-well plates (Corning Costar, Cambridge, MA, USA) coated with 5 μg/mL anti-CD3ε mAb in 200-μL RPMI 1640 medium (Sigma-Aldrich) containing 50 μM 2-mercaptoethanol (Nacalai Tesque Inc., Kyoto, Japan) and supplemented with 10% (*v/v*) heat-inactivated FBS (SAFC Biosciences, Gaithersburg, MD, USA) and 1% (*v/v*) antibiotic-antimycotic solution (10,000 U/mL penicillin, 10,000 μg/mL streptomycin, and 25 μg/mL amphotericin B; Life Technologies). The cultures were incubated in a humidified atmosphere of 5% CO_2_ at 37 °C. After 48 h, the IFN-γ, IL-4, and IL-10 levels in the culture supernatants were evaluated using cytokine-specific enzyme-linked immunosorbent assays (ELISAs) that are commercially available from BD Biosciences [5,6].

### 2.6. Flow Cytometry

Splenocytes and mLNs (2 × 10^6^) were incubated with anti-CD16/CD32 mAb for 20 min on an ice bath. Then, MDSCs were stained with anti-Gr-1 and CD11b mAbs, and the migratory DC were stained with MHC-class II, CD11b, CD11c, and CD103 mAbs. T cells were stained with anti-CD4, CD25, and CD8 mAbs for 30 min on an ice bath, fixed with FACS Lysing Solution (BD Biosciences) for 10 min at room temperature (RT), permeabilized with FACS Permeabilizing Solution 2 (BD Biosciences) for 10 min at RT, and then stained with anti-Foxp3 mAb for 30 min. The stained cells were analyzed using the Accuri™ Flow Cytometer (BD Biosciences) and the FlowJo Software version 9 (Treestar, Inc., San Carlos, CA, USA).

### 2.7. Gut Permeability Test

To establish the gut bacterial leakage model, the mice were administered with 1.5% (wt/vol) dextran sulfate (DSS; MW36,000–50,000; ICN Biochemicals, Costa Mesa, CA, USA) via drinking water at 14 days post-4T1 inoculation. After seven days of treatment, colon tissue and blood sampling were performed [24]. Hematoxylin and eosin (H&E) staining was performed on the section of paraffin-embedded colon tissue as previously described [25]. The serum (1→3)-β-D-glucan level was evaluated using the Fungitec G Test MKII “Nissui” (Nissui Pharmaceutical Co., Ltd., Tokyo, Japan), according to the manufacturer’s instructions [26].

### 2.8. Statistical Analyses

Statistical analyses were performed using the Mann–Whitney *U* test. All analyses were performed using the GraphPad Prism Software Version 6 (GraphPad Software Inc, San Diego, CA, USA). A *p* value < 0.05 was considered statistically significant.

## 3. Results

### 3.1. Intestinal Immunity Is Affected by Subcutaneous Inoculation in 4T1 Tumor

**Figure 1:**
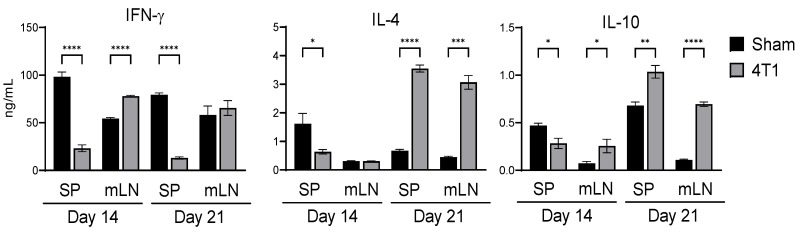
Cytokine production by splenocytes and mLN cells in 4T1 tumor mice. At day 21 of transplantation into the right flank transplantation, the splenocytes and mLNs from control (Sham) or tumor (4T1)-bearing mice were isolated and stimulated with plate-bound anti-CD3ε mAb for 48 h. IFN-γ, IL-4, and IL-10 cytokines in the culture supernatant were detected by ELISA. Data are shown from a representative experiment from four independent experiments. All data are presented as mean ± SEM and assessed with the Mann–Whitney *U* test. * *p* < 0.05; ** *p* < 0.01; *** *p* < 0.001; **** *p* < 0.0001.

To explore the impact of subcutaneous tumors on systemic immunity and intestinal immunity, we created a murine breast tumor model that spontaneously produces a highly metastatic tumor. Tumor volume changes are shown in Supplementary Figure S1. After subcutaneous inoculation of the 4T1 tumor, macro-metastasis was visually observed at day 21 in the spleen, but was not observed in the intestine and mLNs, as is consistent with other studies [19,27]. We initially investigated the cytokine expression of T cells in the spleen and mLNs of these mice ex vivo. IFN-γ, IL-4, and IL-10 production in splenic T cells significantly decreased at day 14, while at day 21, IFN-γ production was suppressed, but IL-4 and IL-10 production significantly increased as compared to control mice (Figure 1). We also analyzed the cytokine expression of T cells in the mLNs. Interestingly, IFN-γ expression significantly increased in 4T1-inoculated mice at day 14, and the expression was equivalent to control mice even at day 21. Increased levels of IL-10 at day 14 was also observed in the mLNs of 4T1-inoculated mice. Furthermore, IL-4 and IL-10 production in 4T1-inoculated mice dramatically increased compared to control mice at day 21. These results indicate that the intestinal immune balance between anti- and pro-inflammatory states changed during tumor development. Since the late stage of breast cancer development generated systemic immune suppression, we further addressed the immune state at day 21 from 4T1 cell inoculation.

### 3.2. Breast Cancer Formation Does Not Affect T Cell Number in mLN

**Figure 2:**
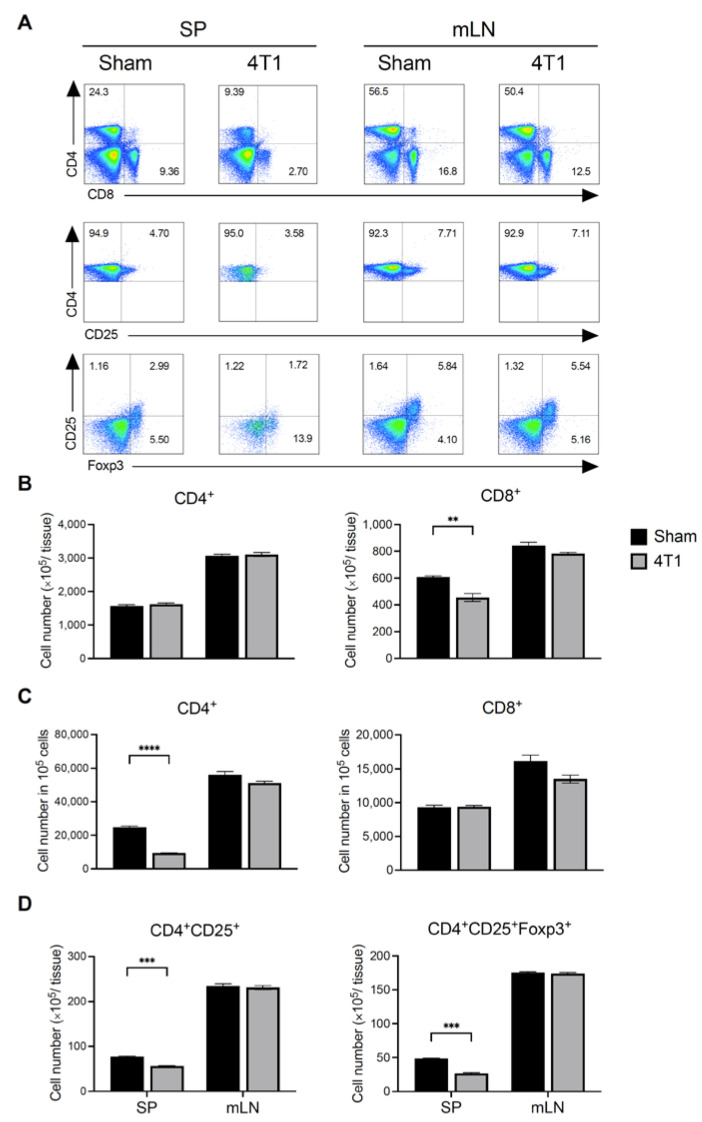
Splenic and mLN T cell subsets in subcutaneously transplanted 4T1 tumor mice. At day 21 after subcutaneous transplantation into the right flank transplantation, the splenocytes and mLN cells from control (Sham) or tumor (4T1)-bearing mice were isolated. (**A**) Representative pseudocolor dot plot of CD4^+^, CD8^+^, CD4^+^CD25^+^, and CD4^+^CD25^+^Foxp3^+^ in the spleen and mLN. Numbers in plots indicate the percentage. (**B**) Total cell number of CD4^+^ and CD8^+^ T cells in the tissues. (**C**) Cell number of CD4^+^ and CD8^+^ T cells per 10^5^ cells. (**D**) Total cell number of CD4^+^CD25^+^ cells and CD4^+^CD25^+^FoxP3^+^ Treg cells in the tissue. Data are presented as mean ± SEM and were assessed by Mann–Whitney *U* test. Data are shown from a representative experiment from three independent experiments. ** *p* < 0.01; *** *p* < 0.005; **** *p* < 0.001.

We next investigated the number of T cells in 4T1 tumor inoculated mice at day 21. Splenocytes and mLN cells were isolated and stained with T cell markers. Both CD4^+^ and CD8^+^ T cells per 10^5^ splenocytes decreased in 4T1 tumor-bearing mice, whereas total splenic CD4^+^ T cells were unchanged (Figure 2A–C). Moreover, the total number of CD4^+^ CD25^+^ T cells and CD4^+^ CD25^+^ Foxp3^+^ Tregs in 4T1 mice was reduced (Figure 2D). In contrast, these cell numbers in 4T1 tumor-bearing mice did not change in the mLN compared with that in sham treatment mice. These results suggest that T cell populations and their cell numbers in mLNs were independent of the decrease in systemic immune cells due to tumor growth.

### 3.3. MDSCs Increased in Spleen but Not in mLN after Tumor Inoculation

**Figure 3:**
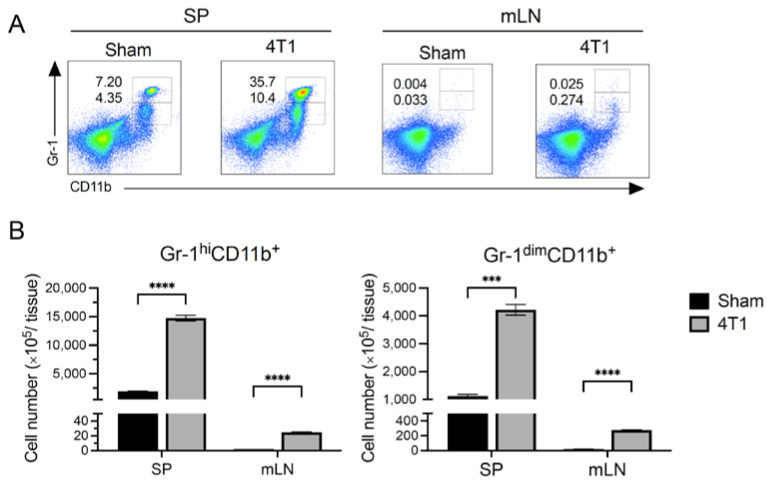
Splenic and mLN MDSC subsets in 4T1 tumor mice. At day 21 of transplantation, the spleen and mLNs from control (Sham) or tumor (4T1)-bearing mice were isolated. (**A**) Representative pseudocolor dot plots and (**B**) cell numbers of Gr-1^hi^CD11b^+^ and Gr-1^dim^CD11b^+^ MDSC subsets. Numbers in plots indicate the percentage. Data are presented as mean ± SEM and were assessed by a Mann–Whitney *U* test. Data are from one experiment representative of four independent experiments. *** *p* < 0.005; **** *p* < 0.001.

To investigate the mechanism by which T cell cytokine responses were altered in the mLN by 4T1 tumor development while T cell numbers were not, we evaluated myeloid cell subsets that influence T cell phenotype and activation at 21 days after inoculation. MDSCs consist of a heterogenous cell population that have the capacity to suppress immunity [28,29]. Thus, we checked the number of MDSCs in the spleen and mLNs of 4T1-inoculated mice. MDSCs were identified as two populations which were marked as Gr-1^dim^ CD11b^+^ and Gr-1^hi^ CD11b^+^ [5,6,30]. The splenic population of MDSCs dramatically increased after 4T1 inoculation (Figure 3). In contrast, the cell number of MDSCs in the mLNs was 50-fold lower than that in the spleen, and this number increased in 4T1-inoculated mice as compared to that in the control (Figure 3). These results suggest that the main cause of splenic T cell reduction was enrichment of MDSCs, but such a reduction of T cells was not predominant in the mLNs after 4T1 inoculation mice owing to the low amount of MDSCs.

### 3.4. Both LN-Resident and Migratory DC Subsets Increase in mLNs of 4T1 Tumor-Inoculated Mice

**Figure 4:**
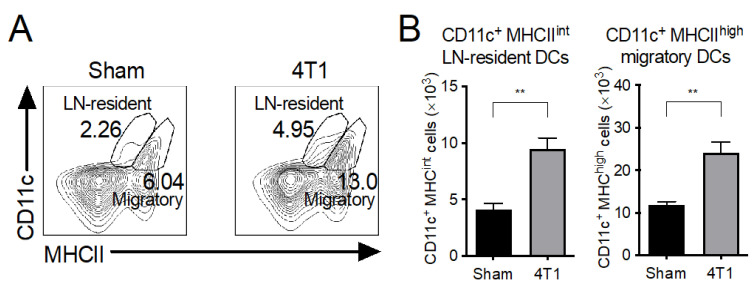
MLN DCs were increased in 4T1 tumor mice. At day 21 of transplantation into the right flank transplantation, mLNs from control (Sham) or tumor (4T1)-bearing mice were isolated. (**A**) Representative flow cytometry plots and (**B**) cell numbers of CD11c^+^MHCII^int^ LN-resident DC and CD11c^+^MHCII^hi^ migratory DC subsets. Numbers in plots indicate the corresponding percentage. Data are shown from a representative experiment from four independent experiments. The mean ± SEM and assessed with a Mann–Whitney *U* test. ** *p* < 0.01.

DCs in the mLN prime and polarize naive CD4^+^ T cells into appropriate effector T helper (Th) subsets, each of which secrete characteristic cytokines [31,32]. There are two main DC populations in the mLN. One is migratory DCs, which are located on the lamina propria in a steady state and can prime effector T cells in mLNs. The other is LN-resident DCs, which are replenished from blood, but their contribution in the priming of naive T cells is still unclear [31,32]. Since the cytokine expression profiles from T cells in the mLNs of 4T1-inoculated mice were altered (Figure 1), we next evaluated these DC populations in the mLNs. LN-resident DCs and migratory DCs are identified by their surface expression of either CD11c^+^ MHC-class II (MHCII)^intermediate (int)^ or CD11c^+^ (MHCII)^high^ (Figure 4A) [33,34]. The cell number of both LN-resident and migratory DC subsets significantly increased in the mLNs of 4T1 tumor-inoculated mice compared to that in the sham control (Figure 4B). We also observed an upregulation of CD80 on DCs in 4T1 tumor-inoculated mice, indicating that DCs in mLNs were activated (Supplementary Figure S2). These results indicate that migratory DCs may contribute to the polarization of IFN-γ-expressing T cells during 4T1 tumor development.

### 3.5. CD103^+^CD11b^+^ Migratory DCs Increase in mLNs of 4T1 Tumor-Inoculated Mice

**Figure 5:**
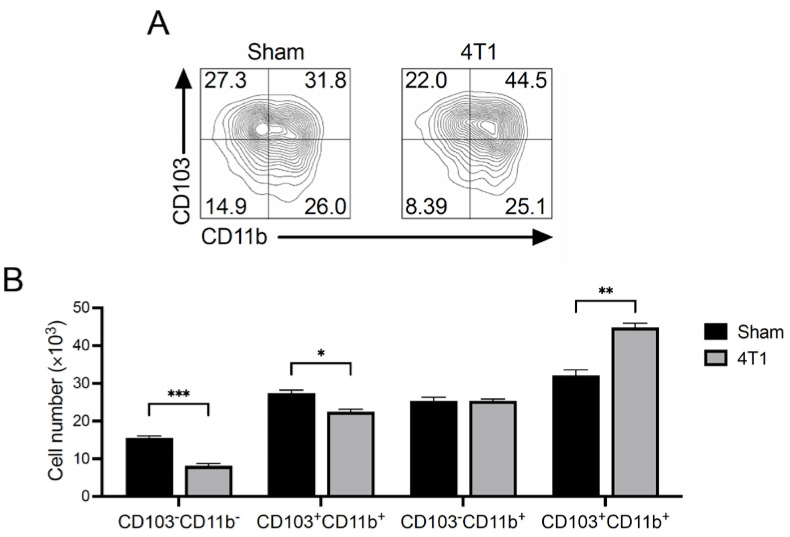
MLN CD103^+^CD11b^+^ DCs were increased in 4T1 tumor mice. At day 21 of transplantation into the right flank transplantation, mLNs from control (Sham) or tumor (4T1)-bearing mice were isolated. (**A**) Representative flow cytometry plots and (**B**) the total number of CD103^+^CD11b^−^, CD103^+^CD11b^+^, CD103^−^CD11b^−^, and CD103^−^CD11b^+^ in CD11c^+^ MHCII^high^ migratory DC subsets. Numbers in plots indicate the percentage. Data are shown from a representative experiment from four independent experiments. The mean ± SEM and assessed with a Mann–Whitney *U* test. * *p* < 0.05; ** *p* < 0.01; *** *p* < 0.005.

CD11c^+^MHCII^high^ migratory DCs can be divided based on the expression of surface integrins CD103 and CD11b. Thus, four subsets of these cells, namely CD103^+^CD11b^−^, CD103^+^CD11b^+^, CD103^−^CD11b^+^, and CD103^−^CD11b^−^ (Figure 5), could be defined. These differ in terms of their ability to prime and polarize different Th cells. Thus, we characterized populations of CD11c^+^MHCII^high^ migratory DCs in the mLNs by evaluating CD103 and CD11b expression [31,34,35,36]. The number of CD103^+^CD11b^+^ DCs was high in 4T1-inoculated mice (Figure 5B). Although the number of CD103^+^CD11b^−^ and CD103^−^CD11b^−^ cells increased significantly in 4T1-inoculated mice compared to sham controls, the increase was lower than that of CD103^+^CD11b^+^ cells (Figure 5B). Intestinal CD103^+^ DCs regulate CD4^+^ T cell fate, including the differentiation into Th1, Th2, and Th17 cells and the development of Foxp3^+^ Tregs [31,37]. These results suggest that intestinal immune homeostasis is disturbed in 4T1-inoculated mice.

### 3.6. Gastrointestinal (GI) Permeability Barrier Defect Increase in 4T1 Tumor-Inoculated Mice

**Figure 6:**
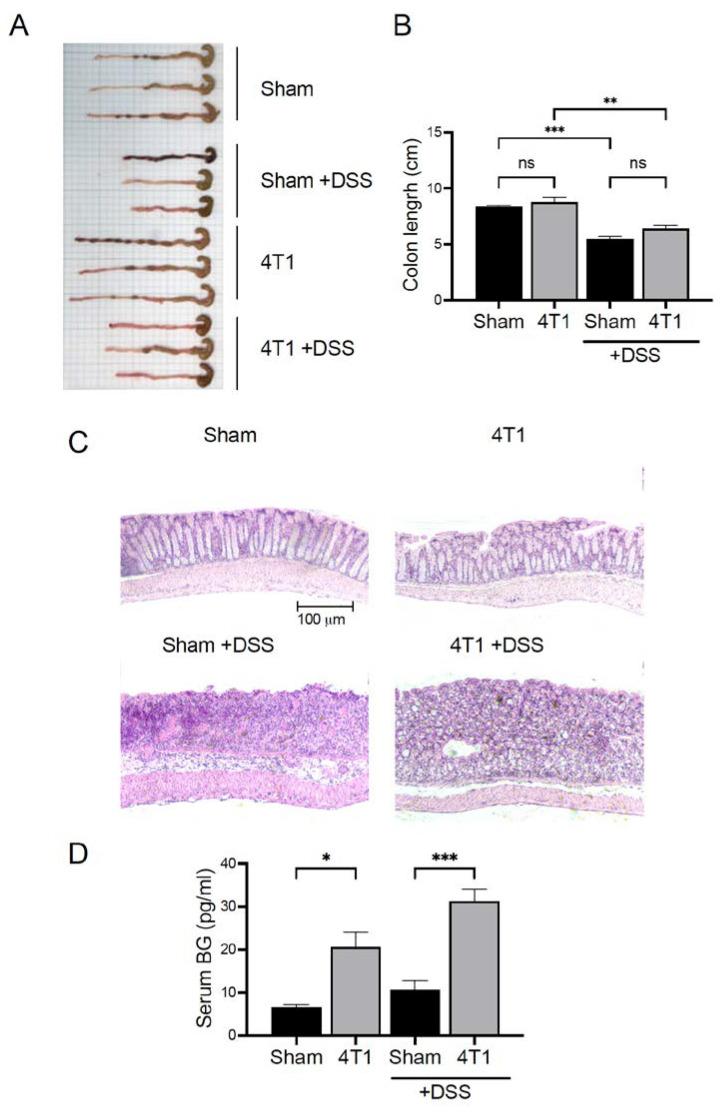
Gastrointestinal permeability barrier defects in 4T1 tumor mice as determined by serum (1→3)-β-D-glucan. 4T1 cells were transplanted subcutaneously into the right flank of a 6-week-old female mouse. (**A**) The picture of colon of the mice at day 21 and (**B**) the bar graph show the colon length. (**C**) H&E histology of the colon tissue sections. (**D**) The serum (1→3)-β-D-glucan levels were detected by ELISA. Data are from one experiment representative of three independent experiments. All data are presented as mean ± SEM and assessed with a Mann–Whitney *U* test. * *p* < 0.05; ** *p* < 0.01; *** *p* < 0.005; ns = not significant.

Finally, we assessed whether the change in the intestinal environment due to 4T1 tumor progression induced bacterial translocation from the intestinal lumen to the circulation. (1→3)-β-D-glucan (BG) is a component specifically present in the cell wall of fungi and bacteria which activates antigen-presenting cells including DCs, and alters intestinal immune cells from steady state to anti- or pro-inflammatory state [26,38]. Spontaneous elevation in serum BG levels represents the disruption of the gut epithelial barrier. Therefore, we evaluated the intestinal epithelial cell condition and serum BG level in 4T1 tumor-inoculated mice. To artificially disrupt the gut epithelial barrier, mice were treated with DSS via drinking water for 7 days. DSS treatment did not affect primary 4T1 tumor growth during the experimental period (Supplementary Figure S3). The colon length of sham and 4T1-inoculated mice was equivalent in the untreated control. DSS treatment did generate shorter colon lengths compared to untreated groups as expected, but the sham and 4T1-inoculated mice in the DSS treated groups were not significantly different (Figure 6A,B). Interestingly, H&E histology of the colons revealed that 4T1-inoculated mice exhibited abnormal intestinal epithelial organization, but this was much less dramatic in DSS-treated groups (Figure 6C). Therefore, during 4T1 tumor development, the gut epithelial barrier of the mice was mildly disrupted. As expected from these results, the serum levels of BG in 4T1 tumor inoculated mice were significantly higher than those in the control (Figure 6D). Even in the mouse DSS model, 4T1 tumor inoculated mice maintained higher levels of serum BG than those in the sham group (Figure 6D). These results suggest that a leaky intestinal barrier was generated by 4T1 tumor development, allowing the leakage of bacteria-derived BG into circulation.

## 4. Discussion

In this study, we investigated immune cell dynamics in the spleen and mLN in a subcutaneously transplanted metastatic 4T1 tumor mouse model to elucidate the systemic immune response during breast cancer development.

The cytokine production profile of T cells in the spleen and mLN was evaluated. At day 14, IFN-γ, IL-4, and IL-10 production of splenic T cells was remarkably suppressed, which was consistent with the findings of our previous study in nonmetastatic CT26 inoculated mice [5,6]. In contrast to splenic T cells, T cells in the mLN at day 14 showed significant upregulation of IFN-γ. This early phase elevation was sustained in the late phase, indicating that a pro-inflammatory environment in intestinal tissue was generated. The cytokine expression dramatically changed with increased IL-4 and IL-10 expression in both the spleen and mLN at day 21, when metastasis was observed in the spleen (Supplementary Figure S4). Of note, IFN-γ expression by T cells in 4T1-inoculated mice was suppressed in the spleen as compared with the sham control, but was equivalent to that in the mLN. Although the effects of IL-4 and IL-10 in breast cancer development are inconclusive toward anti- and pro-tumor effects, late-stage breast cancer patients exhibited high levels of these cytokines in their sera [39,40,41]. Therefore, the high level of IL-4 and IL-10 expressing T cells in the spleen, as well as mLN at day 21, indicates systemic immune suppression at the late stage of breast cancer. 

The number of T cells was significantly reduced in the spleen but not in the mLNs. MDSCs express arginase 1 and inducible nitric oxide synthetase, leading to the suppression of T cell proliferation and function [42]. In fact, the MDSCs in the spleen of 4T1-inoculated mice accumulated more than those of control mice. Although MDSCs in the mLNs of 4T1-inoculated mice were higher than that in control mice as well, the T cell number remained unchanged in the mLNs, as the increase in the number of MDSCs was negligible. This could be a reason why the T cell number was not significantly reduced in the mLNs of 4T1-inoculated mice.

Considering that DCs control T cell polarization, we assessed the number of DCs and their subsets in mLNs. We found significant enrichment of both resident and migratory DCs in mLNs. DCs migrate infiltrating tumor antigens into the lymph node, and both resident as well as migratory DCs can stimulate cancer-reactive T cells [43]. Thus, cancer-reactive T cells might expand in the mLNs. The phenotype of migratory DCs was mainly CD103^+^CD11b^+^. These DCs in the mLN prime naive T cells into effector CD8^+^ T cells, Th cells, and Treg cells [31]. Although how CD103^+^ CD11b^+^ DCs balance Th and Treg cell differentiation is not yet clear, the number of Treg cells did not increase after 4T1 inoculation even at day 21. 

To address how DCs generated IFN-γ-expressing T cells, we evaluated the intestinal environment. The H&E histology showed mildly disrupted gut epithelial cells in 4T1-inoculated mice. Although exactly how the organization of gut epithelial cells was disrupted during 4T1 tumor development is unknown, the leaky intestinal barrier increased the serum BG level in the mice. β-glucan activates DCs by interacting with its receptors, CR3 (CD11b/CD18) and dectin-1, promoting IFN-γ^+^ Th17 and CD8^+^ T cell priming and differentiation [44,45,46]. In addition, oral administration of β-glucan has been shown to enhance antitumor immunity in a mouse tumor model [47]. Consistent with other studies, the DCs of mLNs in 4T1-inoculated mice upregulated the expression of a costimulatory molecule, CD80, suggesting that DCs in the mLNs were activated to promote IFN-γ-expressing T cell priming. Therefore, the CD103^+^CD11b^+^ DCs in the mLNs of 4T1-inoculated mice stimulated by BG might induce IFN-γ-expressing CD4^+^ or CD8^+^ effector T cells, leading to equivalent IFN-γ expression compared to control mice even in the late stage of breast cancer development. 

The depletion of commensal bacteria impacts antitumor immunity. For example, treatment with antibiotics increases the risk of incidence and fatality of breast cancer due to suppressed immune function. It also negatively influences immune checkpoint blockade treatment in cancer patients [48,49]. The depletion of commensal bacteria could have caused a decrease in BG level in such subjects, leading to suppressed immunity. Treatment with antibiotics could contribute to tumor incidence and progression. Therefore, the intestinal immune system with commensal bacteria is important for the optimal functioning of antitumor T cells. Since we found that T cells in the mLN in the early stage of 4T1 tumor development showed pro-inflammatory cytokine profiles, those T cells could protect against breast cancer incidence and development. Furthermore, the T cell cytokine profile in mLNs showed a dramatic conversion from a pro-inflammatory state to an anti-inflammatory state during 4T1 tumor development. Hence, immunotherapy to invigorate cancer-reactive T cells in mLNs in the early stage of breast cancer might result in better chances of success in the treatment.

## 5. Conclusions

In summary, our study demonstrated the dramatic conversion of T cell polarization in the mLN during metastatic breast cancer development. Although systemic immune suppression was observed in the late stage of 4T1 tumor development, the intestinal immune system exhibited several pro-inflammatory characteristics, including accumulation of CD103^+^CD11b^+^ DCs in mLNs, a leaky intestinal barrier, and increased blood BG levels. These results suggest that the intestinal immune system has a considerable effect on the functioning of antitumor immunity during metastatic breast cancer development, which can be further explored to potentially improve cancer treatment by using checkpoint blockade immunotherapy.

## Data Availability

The datasets in this study are available from the corresponding author upon reasonable request.

## Supplementary Materials

The supporting information can be downloaded at: https://www.mdpi.com/article/10.3390/ijms231911035/s1.
