# Supplementary material for: Immune State Conversion of the Mesenteric Lymph Node in a Mouse Breast Cancer Model
Source: Int J Mol Sci. 2022 Sep 20;23(19):11035. doi: 10.3390/ijms231911035 (PMC9570492; doi:10.3390/ijms231911035)
Supplement: Supplementary file 1 [file ijms-23-11035-s001.zip › ijms-1882689-supplementary.pdf]

Supplementary Figure S1

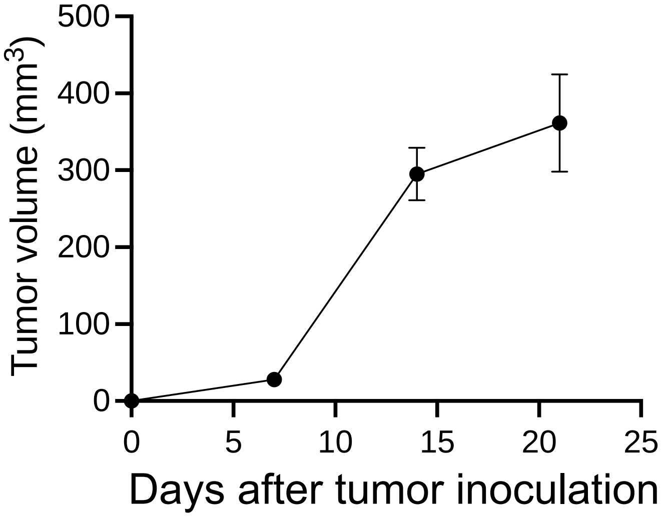

Supplementary Figure S1. Primary tumor growth curve of 4T1 inoculated mice. 4T1 cells were inoculated into the right flank of BALB/c mice. Tumor size was monitored and tumor volume was calculated using following formula; tumor volume = (length x width2)/2.

Supplementary Figure S2

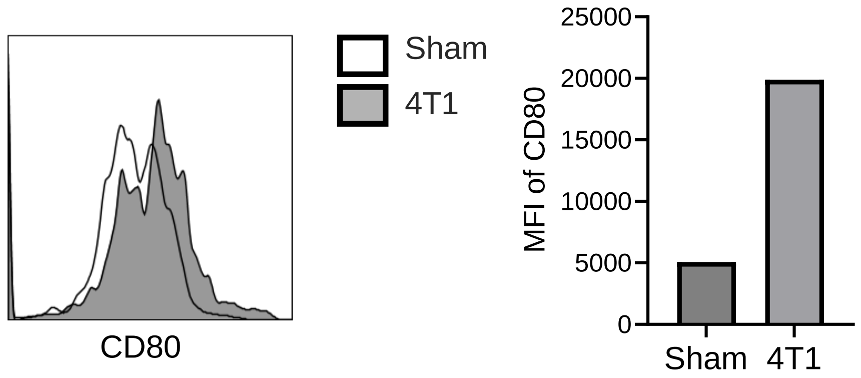

Supplementary Figure S2. Evaluation of CD80 expression on DCs in 4T1 inoculated mice. At day 21 of transplantation into the right flank, mLN from control (Sham) or tumor (4T1)-bearing mice were isolated. Flow cytometry histogram of CD80 in $CD11c^+$ DCs is shown.

Supplementary Figure S3

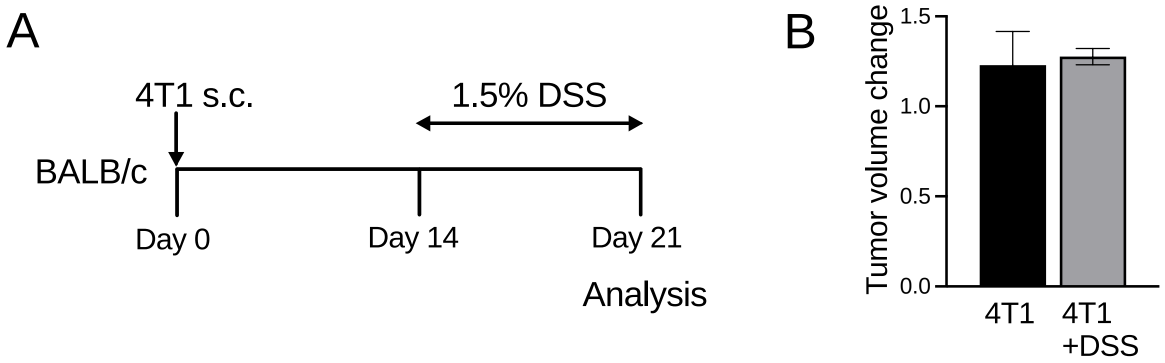

Supplementary Figure S3. Establish the gut bacterial leakage model in 4T1 tumor inoculated mice. (**a**) Experimental scheme. At 14 days of subcutaneous 4T1 transplantation, the mice were treated with 1.5% (wt/vol) DSS in drinking water for 7 days to generate the gut bacterial leakage model. (**b**) Relative tumor volume changes from day 14 to day 21 in 4T1 transplanted mice.

Supplementary Figure S4

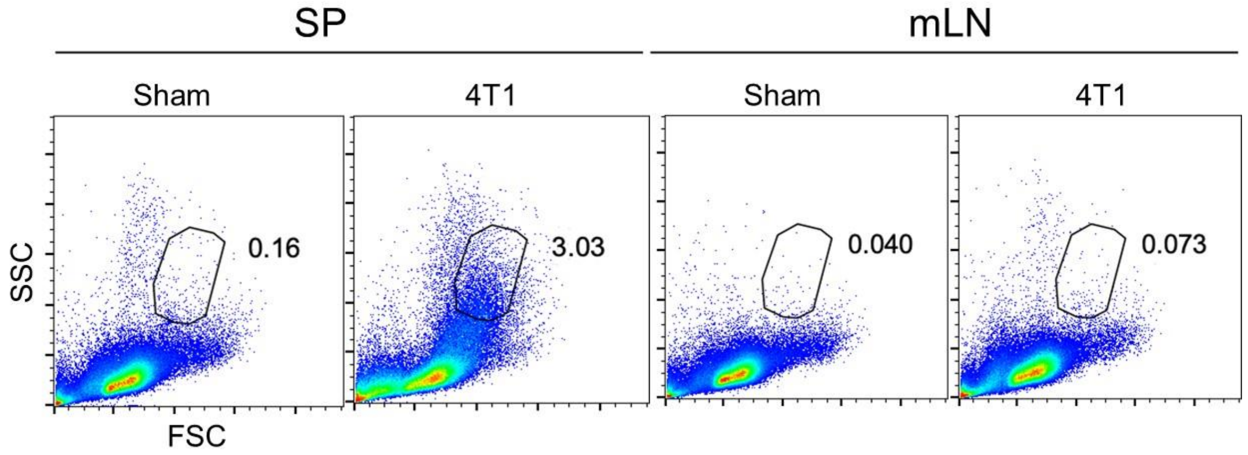

Supplementary Figure S4. FSC/SSC plots of flow cytometric analysis of spleen and mLN in sham and 4T1 tumor inoculated mice. The FSC/SCC plots showed a unique cell population (presumably metastasized 4T1 cells) in spleen of 4T1 tumor inoculated mice.
